# Applying hybrid effectiveness-implementation studies in equity-centered policy implementation science

**DOI:** 10.3389/frhs.2023.1220629

**Published:** 2023-09-08

**Authors:** Yuka Asada, Aimee Kroll-Desrosiers, Jamie F. Chriqui, Geoffrey M. Curran, Karen M. Emmons, Debra Haire-Joshu, Ross C. Brownson

**Affiliations:** ^1^ Community Health Sciences, School of Public Health, University of Illinois Chicago (UIC), Chicago, IL, United States; ^2^ VA Central Western Massachusetts Health Care System, Leeds, MA, United States; ^3^ Department of Population and Quantitative Health Sciences, UMass Chan Medical School, Worcester, MA, United States; ^4^ Department of Health Policy and Promotion, School of Public Health and Health Sciences, UMass Amherst, Amherst, MA, United States; ^5^ Health Policy Research, Institute for Health Research and Policy, School of Public Health, University of Illinois Chicago, Chicago, IL, United States; ^6^ Department of Health Policy and Administration, School of Public Health, University of Illinois Chicago, Chicago, IL, United States; ^7^ Departments of Pharmacy Practice and Psychiatry, Center for Implementation Research, University of Arkansas for Medical Sciences, Little Rock, AR, United States; ^8^ Department of Social and Behavioral Sciences, Harvard T.H. Chan School of Public Health, Boston, MA, United States; ^9^ Department is Public Health, Brown School at Washington University in St. Louis, St. Louis, MO, United States; ^10^ Prevention Research Center, Brown School at Washington University in St. Louis, St. Louis, MO, United States; ^11^ Division of Public Health Sciences, Department of Surgery, Alvin J. Siteman Cancer Center, Washington University School of Medicine, Washington University in St. Louis, St. Louis, MO, United States

**Keywords:** policy implementation science, hybrid effectiveness-implementation, equity, study design, policy research

## Abstract

Policy implementation science (IS) is complex, dynamic, and fraught with unique study challenges that set it apart from biomedical or clinical research. One important consideration is the ways in which policy interacts with local contexts, such as power and social disadvantage (e.g., based on ability, race, class, sexual identity, geography). The complex nature of policy IS and the need for more intentional integration of equity principles into study approaches calls for creative adaptations to existing implementation science knowledge and guidance. Effectiveness-implementation hybrid studies were developed to enhance translation of clinical research by addressing research questions around the effectiveness of an intervention and its implementation in the same study. The original work on hybrid designs mainly focused on clinical experimental trials; however, over the last decade, researchers have applied it to a wide range of initiatives and contexts, including more widespread application in community-based studies. This perspectives article demonstrates how effectiveness-implementation hybrid studies can be adapted for and applied to equity-centered policy IS research. We draw upon principles of targeted universalism and Equity in Implementation Research frameworks to guide adaptations to hybrid study typologies, and suggest research and engagement activities to enhance equity considerations; for example, in the design and testing of implementing strategies. We also provide examples of equity-centered policy IS studies. As the field of policy IS rapidly evolves, these adapted hybrid type studies are offered to researchers as a starting guide.

## 1. Introduction

Policy is a cornerstone of public health interventions, as evidenced by the many policies, such as seatbelt and tobacco laws, that were critical to advancing public health (1). The field of policy implementation science (IS) is distinct from policy implementation research, the latter originated from political science and focuses broadly on how governments put policies into effect (2). Both fields consider the recursive cycles, feedback loops, and processes involved in the policy cycle (3). Policy IS is defined as a field that “seeks to understand how the roll out of policies can be optimized to maximize health benefits” (4); broadly, the field aims to ensure policies are developed with high quality evidence, and/or inform successful implementation of policies once they are codified (4, 5).

Implementation science offers important advancements for policy IS research, which traditionally has measured policy impact or effectiveness (did the policy “work” as intended) with a lighter focus on understanding how, why, and in what contexts? (6). In a review of NIH-funded D&I research, 110 studies were identified that included the term “policy” (or a related term, e.g., law); of those, only 16 studies (14.5%) examined factors or mechanisms of implementation, or tested strategies to improve policy implementation (7). A better understanding of implementation outcomes, processes, contexts, and determinants of policy implementation allows us to discern whether the observed organizational, health, or behavioral outcomes are a result of the policy or in fact are artifacts of incomplete or poor implementation.

Policy IS inquiries may draw from effectiveness-implementation hybrid studies, which were developed to address research questions around the effectiveness of an intervention and its implementation in the same study (8). This original work was mainly focused on clinical experimental trials; however, researchers have applied it to a wide range of interventions and contexts, including more widespread application in community-based studies of evidence-based interventions (EBIs) (9). While typologized in nomenclature, the hybrid study types are more of a continuum than distinct categories, with a focal decision point being the level of “evidence” available about the “thing” or intervention of interest (see discussion on the “thing” below) (10, p. 2). Typically, a researcher may consider starting with a Type 1 study when there is less data on intervention effectiveness, to understand its effectiveness while understanding the context for implementation; Type 2 focuses on collecting intervention effectiveness data but also moves simultaneously toward understanding feasibility/utility of an explicit implementation strategy (either alone or comparatively) to support delivery of an intervention; and Type 3 determines utility of (two or more) implementation strategies and also collects intervention effectiveness data but as a secondary outcome category (9). Hybrid studies allow policy IS research to advance an understanding of how policy is a critical public health tool, while gathering important contextual implementation data to inform uptake in other settings.

The field of implementation science has increasingly called for a greater attention to the intersection of health equity and implementation (4, 5, 12–16). Notably, some research may centrally feature equity while others may not; at minimum, researchers are urged to “leave no one behind” by being intentional about the potential to exacerbate inequities (16). This is notable with policy IS research which is dynamic and fraught with unpredictable real-world events, politics, and ideology (5, 17, 18). Adding to the complexity is an important consideration for the ways in which policies interact with local contexts, including power and social disadvantage (e.g., based on ability, race, class, sexual identity, geography and many others; hereafter: historically disadvantaged groups/communities) (5). Historically disadvantaged communities may not have the resources to fully adopt or implement a policy (19–21). For example, the 2006 Massachusetts statewide universal health care law expanded access to health insurance for all state citizens; however, after implementation, 96% of non-Hispanic white citizens were insured, compared to only 79% of Hispanic citizens (22). Although Hispanic groups saw an increase in coverage, those with limited English proficiency faced enrollment barriers; also, communities with poorer access to primary care physicians also faced access barriers. Limited attention to the unique needs of historically disadvantaged groups led to exacerbation of racial disparities in health coverage (22).

In this paper, we discuss how effectiveness-implementation hybrid studies can be applied to equity-centered policy IS research (23). We focus on big “P” policy (hereafter, policy) defined as laws, ordinances, rules, regulations, executive orders and court decisions that are enacted by federal, state, or local governments; we do not include small “p” policies (defined as organizational policies and guidelines that are typically not required by laws/regulations from governments) (24) due to distinct implementation factors. We also focus on the study of policies after they are passed, rather than indirectly informing policy development, awareness or adoption, while recognizing that this is an iterative, non-linear, and often dynamic process (4).

## 2. Considerations for policy IS hybrid studies in equity-centered research

### 2.1. Conceptualization of policy as the “intervention” or “thing” of interest

Historically in public health research, policy was more typically conceptualized as a distal “outer setting” determinant and not as the central “intervention” [i.e., as described by Curran using plain language as, “the thing being implemented” (4, 6, 7, 24)]. Policy can be conceptualized as the “thing” of interest, or an “implementation strategy,” (i.e., as described by Curran, “the stuff we do to try to help people and/or places to do the thing”) (24), or a determinant that influences the implementation of strategies (6). For example, school nutrition standards intend to decrease consumption of sugary, low-nutrient foods and beverages. In this case, the “thing being implemented” or the intervention is the policy that intends to make healthier foods and beverages more accessible to students in the school built environment. In comparison, earmarked taxes, defined as “taxes for which revenue can be spent only on specific activities” are conceptualized as an implementation strategy that facilitates access to evidence-based practices, such as mental health services (25). For the policy of interest, articulating clearly its place as “the thing” or an “implementation strategy” early in study conception is critical to the selection of frameworks, study designs, and associated methods (6). We contend that it matters less how it is conceptualized; rather, the important point is that it is clearly described.

### 2.2. Centering equity in policy IS research

Two frameworks: (a) Equity in Implementation Research (EquIR); and (b) targeted universalism inform this work. Briefly, EquIR aims to address inequalities during implementation and was selected because it calls for an explicit and intentional focus on equity—particularly on social determinants of health—from the planning and design phases (23). The framework encourages researchers to consider a continuum of participatory approaches that center historically disadvantaged groups' priorities. Such efforts will avoid constructing historically disadvantaged communities as “homogenous groups with static traits and shared beliefs” (26). Targeted universalism is defined as “pursuing targeted strategies that respond to the urgent needs of some people, and wrapping those strategies in a universal goal that holds wide appeal” (22). This framework was selected because it offers an equity-driven focus to policy strategies and aligned with approaches to health; for example, goals may include providing food, housing, and affordable health care, and the “targeting” component involves measuring the impediments to filling gaps, not with reference to each other but to the universal goal (22). The examples described next are designed with the principles of both EquIR (intentionally building equity into study approaches) and targeted universalism (developing equity-driven policy strategies that promote structural change). Finally, an essential component of equity-centered work is the need for researchers' to deeply engage in reflexive practices (27). Reflexivity requires researchers to continuously check their own social positions and deeply examine the ways in which they exercise and are influenced by power, as well as the ways in which these positions influence the particular research subject (27). While this practice should be conducted in all research, policy is fraught with ideology and values; thus, researchers should be mindful and transparent about their own biases and privileges.

### 2.3. What are policy and equity goals?

**Figure 1:**
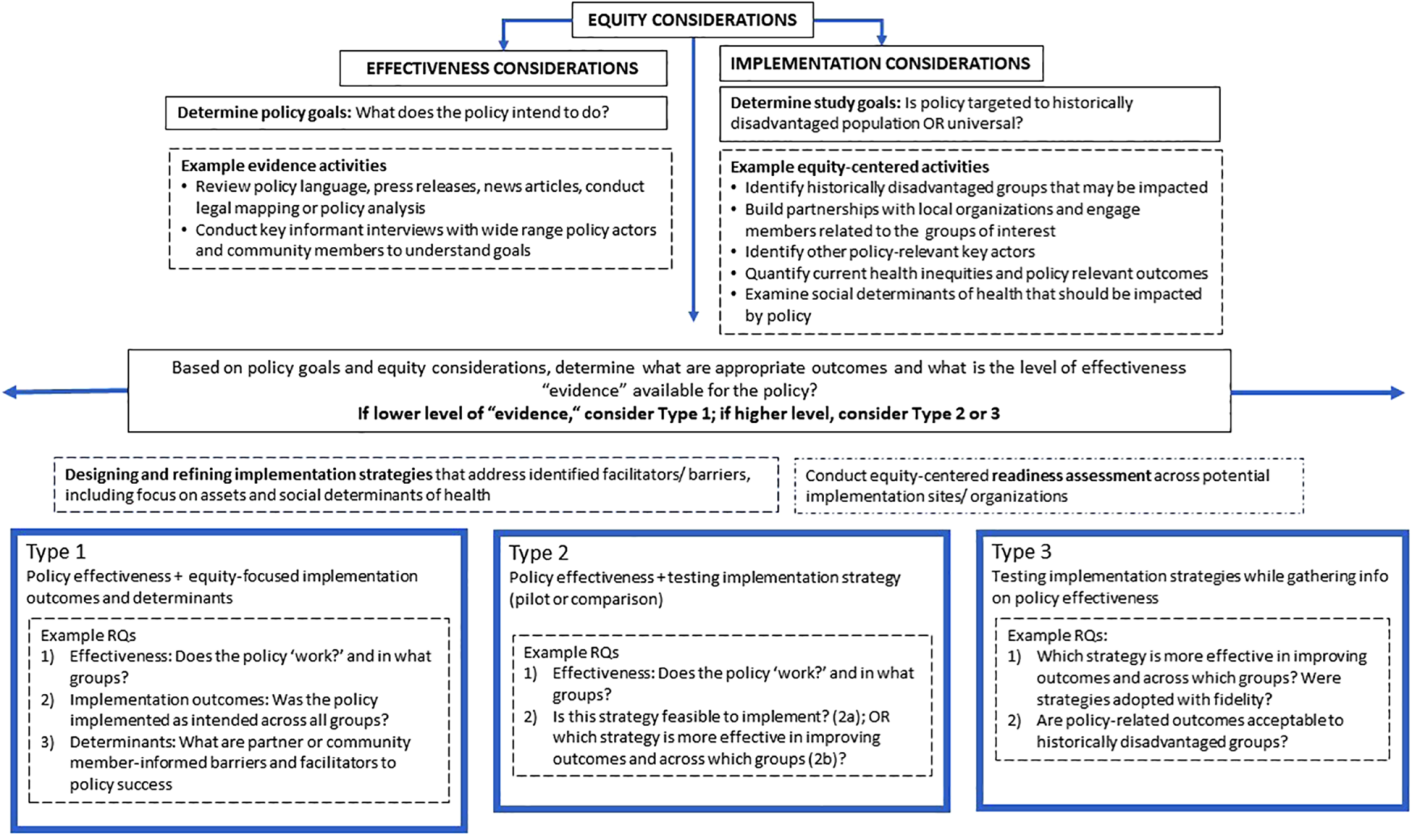
Considerations for equity-centered policy implementation science hybrid studies.

Figure 1 illustrates two key considerations. First, at the top of the figure, researchers are reminded to consider equity early in the design phase and across effectiveness and implementation considerations. Second, to address “effectiveness,” researchers may consider what the policy intended to do and identify the appropriate “evidence” (described next) to support success, failure, or other (6). Unlike clinical interventions, policy as an “intervention” can be ambiguous (i.e., unclear policy language or multiple goals); in response, researchers may conduct (pre-study or phased) activities to better understand policy goals, such as policy analysis (28) or qualitative interviews with key policy actors and community groups (6). Policy analysis may be part of a policy surveillance, the latter is defined as “the ongoing, systematic collection, analysis, interpretation and dissemination of information about a given body of public health law and policy” (28).

We offer a few dimensions of “evidence” to inform policy success or failure. Scholars have outlined the problematic nature of applying post-positivist, clinical hierarchies of “evidence” (for example, based on large, randomized samples, and controlled for confounding variables) to the study of public health policy (3, 29). This discussion sits within a broader conversation and advocacy toward transforming the ways that “evidence” on policy effectiveness is generated and disseminated for decision making (3, 30). Policy “evidence” requires broader data that is derived from multiple sources/actor groups, tailored to context, and responds to the interests of those impacted by the policy (3, 30). Parkhurst and Abeysinghe (2016) suggest considering key questions: “(1) what are the policy concerns at hand (and is the evidence selected the most useful to address the multiple policy concerns at hand)?; (2) are the data constructed in ways that best serve policy goals?; and (3) do we have reason to believe that the evidence is applicable to our local policy context?” (29). Finally, and importantly, we highlight the equity dimension: “evidence” generation has historically been the privilege of white, Western, male researchers, with the intentional exclusion of historically disadvantaged groups, including women and people of color (27, 28). Here again, inclusion of a broader range of outcomes as “evidence,” such as nonbiomedical outcomes more salient to communities of interest is paramount (27). Researchers may determine implementation considerations with an equity lens by identifying who the policy will impact (i.e., is it targeted to a specific historically disadvantaged group or group with documented disparities?) and whether the current “evidence” points to any existing disparities or differential outcomes across groups (23). We restate here that policy “evidence” without this equity lens can unintentionally lead to exacerbation of inequitable conditions (3, 21).

### 2.4. What are policy-specific implementation outcomes?

In addition to investigating outcomes to better understand policy effectiveness (e.g., health or behavioral outcomes), hybrid studies examine implementation outcomes, such as acceptability, feasibility, sustainability and costs (31). Previous scholars have adapted these definitions and identified quantitative measures (outcomes and determinants) for policy IS research (23); for example, acceptability may measure perceptions of historically disadvantaged groups to understand if the policy is “agreeable” and why/why not. Implementation cost may measure total costs of implementation for historically disadvantaged and non-disadvantaged groups and calculate a final adjusted cost-effectiveness (23). More work is needed to further test and develop psychometric properties of such tools, as well as ensure rigorous qualitative and mixed methods approaches to measuring outcomes (23).

## 3. A continuum of hybrid studies for equity-centered policy IS

While not distinguished as a hybrid type, for illustrative purposes, we consider a non-hybrid Type 0 example, which is akin to those typically conducted in health policy studies, and examines effectiveness outcomes after a policy is adopted (e.g., did the smoke-free policy reduce smoking in the jurisdiction?) without measuring implementation outcomes (e.g., did the jurisdiction implement the smoke-free policy as intended?) or contextual factors (e.g., what factors led to policy success or failure?) (32). Type 0 studies do not elucidate whether some groups implemented the policy more effectively and/or benefited disproportionately. We include this example to illustrate the status quo from which these proposed hybrid approaches depart.

### 3.1. Hybrid type 1

Table 1 provides key characteristics of each hybrid type to align with policy IS research. Again, they are presented as distinct types here, but can be conceptualized more as a continuum. As illustrated in Figure 1, Hybrid Type 1 is considered when there is the lowest availability of “evidence” on whether the policy is effective (9), while considering whether outcomes differ in historically disadvantaged groups: if the policy “works,” did it “work” across all groups? For example, in the case of sugary beverages taxes, a policy goal may be a decrease in purchasing of sugary beverages, based on prior research that has shown differential rates of exposure to targeted marketing across ethnic groups (33, 34). In addition to behavioral outcomes at the individual level, studies may consider policy-level outcomes, such as revenue generated by the tax (35), and community-level outcomes, such as the number of community organizations offering tax-funded programs. A secondary aim is to understand why (or why not) the policy “worked”, including equity-focused implementation outcomes, may examine fidelity to the policy (e.g., were all groups of interest within the jurisdiction able to implement the policy as intended?), as well as perceptions of acceptability of the policy among historically disadvantaged groups. A tertiary aim for the Hybrid Type 1 approach is to understand whether there were unique barriers or facilitators within historically disadvantaged groups that impacted implementation. Addition of contextual and determinant factors allows for a comprehensive understanding: what unique assets facilitated implementation; what were barriers that could inform future implementation strategies? (36).

**Table 1:** Hybrid approaches for policy IS studies with an equity emphasis [adapted from Curran et al. (8)].

	Hybrid type 1 Policy effectiveness and implementation determinants + outcomes	Hybrid type 2 Policy effectiveness + implementation strategy feasibility	Hybrid type 3 Comparing implementation strategies + policy outcomes
Research aims	Primary aim: determine if policy is effective across groups, including historically marginalized groups; Secondary aim: determine policy implementation outcomes, including whether the policy was implemented as intended; Tertiary aim: determine if there are unique facilitators/barriers, including focus on assets and structural factors supporting/impeding implementation for historically marginalized groups	Co-primary aim: determine if policy is effective across groups, including socially minoritized groups; Co-primary aim: 2a. Determine if an equity- focused implementation strategy is feasible *or* 2b. Compare which equity- focused implementation strategy is most effective + implementation outcomes	Primary aim: compare which equity-focused implementation strategy is most effective Secondary aim: gather policy-related outcomes that are community- and/or partner-centered
Sample research questions	Effectiveness: is the policy effective and how do expected outcomes differ across historically marginalized groups? Implementation outcomes: was the policy implemented as intended across all groups/settings? Determinants: what factors led to success or failure of the policy; and do historically marginalized communities experience unique barriers/facilitators	Effectiveness: is the policy effective and how do expected outcomes differ across historically marginalized groups? 2a. Pilot strategy: is a pilot strategy feasible in historically marginalized groups? What is readiness to implement the implementation strategy? 2b. Comparing (two or more) strategies: which strategies best facilitate implementation of policy, and across which historically disadvantaged groups?	Comparing (two or more) strategies: which strategies best facilitate implementation of policy, and across which historically disadvantaged groups? Policy outcomes: are policy outcomes acceptable to historically marginalized groups?

### 3.2. Hybrid type 2a—pilot

Type 2a approaches aim to understand policy effectiveness and pilot test a potential implementation strategy. There may be some evidence to support the policy but effectiveness data is still of interest. A co-primary aim is to understand whether the policy showed differential effectiveness across groups, which requires a baseline understanding of the existing historically disadvantaged groups and potential disparate health status. In addition, a co-primary aim for Type 2a is to test the feasibility of an implementation strategy and “preliminary effectiveness” of the strategy on implementation outcomes (e.g., adoption or fidelity); the latter could be part of a readiness assessment to understand whether historically disadvantaged groups and partners are ready to adopt the strategy of interest. Conducting a rigorous readiness assessment—with an explicit equity emphasis—is considered an important strategy for policy IS research.

### 3.3. Hybrid type 2b—comparison

Type 2b approaches may be considered when there is interest in comparing two (or more, including packages of) implementation strategies in their ability to facilitate implementation, along with implementation outcomes, such as adoption and fidelity. Like Type 2a, a co-primary aim is to measure effectiveness of the policy amongst the historically disadvantaged groups and/or across all groups of interest. Another co-primary aim is to compare effectiveness outcomes between the two (or more) implementation strategies. For example, the same sugary beverage tax study may compare two implementation strategies: (1) a retailer education program to improve knowledge of sugary beverage among retailers; versus (2) a random check monitoring strategy that checks compliance to sugary beverage tax, to determine which of these strategies was more successfully adopted with fidelity. This type of examination may also use a commonly applied framework—such as RE-AIM—to evaluate equity-centered implementation outcomes associated with each implementation strategy, such as how many and what types of retailers received the trainings, to understand if there was differential uptake across retailers and why (37).

### 3.4. Hybrid type 3

Type 3 studies are recommended when there is substantial “evidence” available supporting the effectiveness of the policy (e.g., a systematic review). Pilot testing of implementation strategies—including readiness assessment—would already be completed via a partner-informed approach. Like Hybrid Type 2b, the primary aim is to understand which of the implementation strategies (or packages) “worked” better in facilitating implementation of the policy (comparison of strategies). A secondary aim is to also gather policy effectiveness outcomes (as described in Type 1) to determine if there was success, including in historically disadvantaged groups.

### 3.5. Equity-centered implementation strategies

Figure 1 includes the design or refinement of implementation strategies across the continuum of types as an equity-centered activity. This process aligns with a “targeted universalism” process, where targeted strategies—not a “one size fits all”—are designed based on partner-informed data (22). There are several compilations of general (e.g., Expert Recommendations for Implementing Change (ERIC), school [e.g., School Implementation Strategies, Translating ERIC Resources (38)] and policy-specific implementation strategies [e.g., Bullock et al. (17)] available. In addition, once implementation strategies are identified, a partner-engaged readiness assessment is recommended to understand whether the target partners have the capacity and motivations to implement (39). Many of the processes used in designing for dissemination (e.g., stakeholder engagement, participatory codesign, context analysis) will facilitate partner-focused implementation (40).

## 4. Discussion

To our knowledge, this is the first paper that has described application of hybrid approaches for policy IS research. This paper is intended to be a starting point for discussion, particularly for the ways in which equity can be addressed in examination of policy implementation. Given the potential for policy IS research to advance public health on a population level, we strongly advocate that policy IS studies devoid of an equity approach provide a rationale for the omission.

We offer additional considerations toward this work. First, these typologies do not dictate the research study design; a wide range of designs (e.g., interrupted time series, mixed methods evaluations) may fit and importantly, should be dictated by research questions (9). Although hybrid studies were designed with experimental designs in mind (9), a policy-focused Type 1 hybrid study likely will apply an *observational implementation-effectiveness* hybrid approach since policies do not lend themselves to randomization in experimental trials, particularly studies including social determinants to health (36). Guidance is available for applying hybrid types to observational studies that are particularly salient for policy-focused implementation research; for example, studies may apply quasi-experimental or natural experiment designs that leverage existing or routinely collected (individual or aggregate) program or administrative data (36). Type 2 and 3 studies that test and compare implementation strategies may lend themselves to prospective, experimental studies. In selecting study designs for policy-focused implementation science work, researchers highlight the need to balance the goals of academic rigor with partner- and community-members' capacities and willingness to participate (7).

Second, the examination of policy implementation is necessarily complex and fraught with feedback loops, (un)intended outcomes and consequences due to political, economic, and social inequities (3, 4). Figure 1 is necessarily simplistic. For example, a policy IS hybrid study that examines implementation of a smoke-free public housing policy requires an understanding of social determinants of health—such as access to safe housing, environmental exposure to toxic chemicals—and the impacts of structural racism on policy implementation. The study may intersect with housing, health, and policy sectors, along with a wide range of policy actors and community groups (e.g., public housing residents). Researchers are required to manage the complexity of these multi-level determinants, intersecting sectors, and potential (un)intended outcomes that will shape the research findings (3).

Lastly, policy IS studies can contribute best to health equity by elucidating which policies have the maximum impact on structural support and social determinants of health (5). To this end, instrument development for policy specific implementation outcomes is needed, currently there are some tools for school settings (41, 42) and more broadly (31); however development and testing—including for qualitative data—remains nascent. In addition, examples of policy-specific implementation strategies are organized by target organizational level (e.g., educational trainings) vs. policy authority level (e.g., appointment of state leaders to garner resources) (17). Not captured in these examples but are important considerations include: small “p” policies as an important space to examine equity; as well as when implementation strategies are best targeted to the policy cycle (e.g., exploration, preparation, initial implementation, full implementation, and sustainment) (17). Further work is needed to build the body of literature examining both policy-related outcomes and implementation strategies (6, 32).

## Data Availability

The original contributions presented in the study are included in the article/Supplementary Material, further inquiries can be directed to the corresponding author.
